# Editorial: Research challenge on slip prevention measures in the workplace

**DOI:** 10.3389/fpubh.2022.1105047

**Published:** 2022-12-13

**Authors:** In-Ju Kim

**Affiliations:** Department of Industrial Engineering and Engineering Management, College of Engineering, University of Sharjah, Sharjah, United Arab Emirates

**Keywords:** fall-related injuries, fall-related fatalities, slip resistance measurements, slip and fall incidents, slip and fall safety

## 1. Overview

Slips, trips and falls are amongst the most common causes of injuries and fatalities in the general community and industry. Reducing such incidents involves a complex array of factors including the characteristics of each individual's footwear, gait dynamics, walking and working surfaces, environmental conditions, etc. Notwithstanding this complexity, slip resistance properties have been widely measured as a form of coefficient of friction (COF) index at the sliding interface amongst the shoes, floors, and environments (1). Since COF measurements were commonly adopted to evaluate slip potentials, there have been continuous debates in the assessments and interpretations of COFs (2). This Research Topic for Frontiers in Public Health is principally focused on broadening the knowledge base and developing new concepts and ideas on which improvements in the validity and reliability of slip resistance measurements might be made. To achieve this goal, this Research Topic covers extensive themes on industry/public falls safety developments such as:

1) Engineering/tribology approach to measuring slip resistance properties.

2) Slip resistance (anti-slip) properties of floor/walkway surfaces.

3) Controls of contamination, cleaning, and maintenance.

4) The function of footwear and slip-resistance shoes.

5) Preventing slips, trips, and falls.

This Research Topic also welcomes papers on all other areas of fall prevention and related topics, including gait dynamics and slip resistance, biomechanics and friction controls, falls from heights, stair falls, etc.

Four guest co-editors have worked together to address these issues from inter- and multi-disciplinary perspectives that reflect an appreciation of the biomechanics, clinical, engineering, rehabilitation, and safety milieus in which slips and falls occur. To exemplify this communal approach to the prevention of fall incidence, embodied within this Frontiers Research Topic are four articles enfolding four interrelated topical areas: community-based interventions; biomechanics, engineering, and rehabilitation vistas.

## 2. Participated studies

### 2.1. Kinect-based tailored interactive fall intervention system

This article introduces a novel development of a Kinect-based tailored interactive fall intervention system, which seamlessly integrates multifactorial fall risk assessment and tailored intervention programs to prevent falls in older people. This preliminary study examined the effectiveness and usability of the intervention system for fall prevention in older people (Kim and Xiong). Thirty community-dwelling older women participated in this experiment and were allocated to an intervention group (IG) or a control group (CG) for a quasi-randomized trial (15 people each). The participants in IG followed an 8-week tailored intervention (40 min/session × 2 sessions/week × 8 weeks) using the Kinect-based interactive fall intervention system, whilst the participants in CG maintained their habitual activities. Different outcome measures were evaluated at baseline (Week 0), interim (Week 4), and post-intervention (Week 8). Their results showed that IG led to significant improvements in TUG-Timed Up and Go (*p* = 0.010), BBS-Berg Balance Scale (*p* = 0.011), and Montreal Cognitive Assessment-MoCA (*p* = 0.022) between baseline and post-intervention. In comparison to the baseline, TUG and BBS were even significantly improved in the interim (*p* = 0.004 and 0.047, respectively). There were no significant changes in static balance-related performance outcomes and the Short Falls Efficacy Scale-SFES after the intervention. Whereas, in CG, most performance measures did not show significant changes during the 8 weeks period, TUG completion time became significantly longer at post-intervention in comparison to interim (*p* = 0.028) and fear of falling was also significantly higher at post-intervention than baseline (*p* = 0.021). Findings from this study suggest that the Kinect-based 8-week tailored interactive fall interventions effectively improved older people's physical and cognitive abilities. Regarding the usability of the developed system, the average System Usability Scale (SUS) score was 83.5 out of 100, indicating excellent system usability. The overall mean Computer Literacy Scale (CLS) score was 2.5 out of 26, presenting that older participants in this study had very limited experience with computers. No significant correlation between the SUS and CLS scores demonstrated that the newly developed Kinect-based tailored interactive fall intervention system was easy for older people, regardless of their computer experience. This novel system should help health professionals and older people proactively manage the risk of falls.

### 2.2. Linear discriminant analysis models to discriminate slip-related falls

This article compares the accuracy of four linear discriminant analysis models informed by predetermined sagittal or frontal plane measurements from the lower body (feet velocities relative to the center of mass) or upper body (angular momentum of trunk and arms) during reactive responses after slip initiation. Ouattas et al. challenged in their study to identify these critical kinematic factors due to the wide variety of upper and lower body postural deviations that occur following the slip, which affects stability in both the sagittal and frontal planes. To explore the utility of kinematic measurements from each vertical plane to discriminate slip-related falls from recoveries, we compared the accuracy of four Linear Discriminant Analysis models informed by predetermined sagittal or frontal plane measurements from the lower body (feet velocities relative to the center of mass) or upper body (angular momentum of trunk and arms) during reactive responses after slip initiation. Unconstrained bilateral slips during over-ground walking were repeatedly administered using a wearable device to 10 younger (24.7 ± 3.2 years) and 10 older (72.4 ± 3.9 years) adults while whole-body kinematics were measured using motion capture. Falls (*n* = 20) and recoveries (*n* = 40) were classified by thresholding the dynamic tension forces measured in an overhead harness support system and verified through video observation. Frontal plane measurements of the peak feet velocities relative to the center of mass provided the best classification (classification accuracy = 73.3%), followed by sagittal plane measurements (classification accuracy = 68.3%). Measures from the lower body resulted in higher accuracy models than those from the upper body, but the accuracy of all models was generally low compared to the null accuracy of 66.7% (i.e., predicting all trials as recoveries). Ouattas et al. suggest that future work should investigate novel models including potential interactions between kinematic factors. The performance of lower limb kinematics in the frontal plane in classifying slip-related falls demonstrates the importance of administering unconstrained slips and measuring kinematics outside the sagittal plane.

### 2.3. Consumer perspectives on grab bars

This article characterizes Canadian public perceptions on the installation and use of grab bars in home bathrooms. Given the prevalence and severity of bathroom falls and injuries across age groups, there is growing interest in policy-level approaches to bathroom fall prevention. Grab bars reduce fall risk during bathing transfers and improve bathing accessibility for adults of all ages and abilities. However, according to Levine et al., grab bars are frequently absent from bathing environments, even in the homes of individuals who need for a grab bar. Whilst mandatory bathroom grab bar installation has been suggested, it is unclear whether this would be supported by Canadians. Levine et al. surveyed 443 Canadians about whether they currently had a grab bar and their perspectives on the grab bar policy. Survey results show that 65.4% of the respondents did not have a grab bar whilst 88.5% would allow a grab bar to be installed in their bathroom at no cost to them, only 11.5% of respondents would object to grab bar installation becoming mandatory in new builds, and 85.6% of respondents would use a grab bar if it were installed in their bathroom. Responses were affected by age (in four groups: 18–39, 40–59, 60–79, and 80+ years), self-reported impairment, and home ownership status. Older adults, who reported having impairments, and homeowners were more likely to respond favorably toward grab bars. Based on these results, this study concluded that the majority of Canadians would respond positively to a policy mandating bathroom grab bars in new homes.

### 2.4. Contaminant film thickness affects walkway friction measurements

This article investigates the effect of contaminant film thickness against walkway friction measurements. Chimich et al. used walkway tribometers to measure available friction to evaluate walkway safety and pedestrian slip risk. Because numerous variables can affect tribometer measurements, including the type and distribution of contaminants on the surface, they quantified the effect of the application method on contaminant film thickness, and the effect of film thickness on tribometer measurements from the four reference walkway surfaces according to ASTM F2508-16e. To quantify naturally occurring film thicknesses, they exploited distilled water, 0.05% sodium lauryl sulfate (SLS) solution, and 0.04% Triton X-100 solution onto the surfaces. These application methods had a significant effect on the resulting film thickness (*p* < 0.038) with the pour method consistently generating the thickest films and the spray method generating the thinnest films. Chimich et al. then quantified the effect of film thickness for the three contaminants (thickness range 0.3–3.3 mm) on the friction measurements of three common tribometers (Mark IIIB, English XL, and BOT 3000E) on each reference surface. A separate ANOVA was compared for each of 36 combinations (3 × 4 × 3) of tribometer, surface, and contaminant. This study found that friction measurements with the Mark IIIB decreased with increasing film thickness on one surface across all three contaminants and on a second surface with the SLS contaminant. Friction measured with the BOT 3000E was sensitive to film thickness on two surfaces with water and one surface with Triton. Measurement results from the English XL were unaffected by the contaminant film thickness. Overall, despite significant differences in film thickness with contaminant application methods, friction measurements were either insensitive to film thickness or varied only a small amount in all cases except for the Mark IIIB on the roughest surface. Film thickness did not alter the relative slip resistance of the four ASTM F2508 reference surfaces.

## 3. Conclusion

Fall incidents from slips or trips have been recognized as a major safety and health issue in the industry and daily living. They represent the second leading cause of accidental deaths after motor vehicle accidents so preventing falls is a great issue in organizations and communities around the world. As a result, prolonged efforts have been poured in to apprehend the vital causes of such incidents. The etiology of falls as injury-producing events is multifactorial and comprises numerous mechanisms of exposure. To tackle the innumerable causes of multifactorial events, there require to be wide-ranging, inter- and multi-disciplinary approaches, and translational efforts to mitigate and provide concerned parties with validated research findings, methods and recommendations for safe practices. In this context, this Research Topic “*Research Challenge of Slip Resistance Measurements*” grew out of intense discussions on the topic held from a perspective of various approaches. Although significant challenges and questions continue to explore, the findings presented in this Research Topic suggested ample arrays of research approaches, research findings, recommendations, and expert advice on the latest tools and methods to reduce the incidence of injury from falls.

## Author contributions

The author confirms being the sole contributor of this work and has approved it for publication.
